# Conscious breathing enhances bidirectional cortical-autonomic modulation: dynamics of EEG band power and heart rate variability

**DOI:** 10.3389/fnsys.2025.1650475

**Published:** 2025-09-29

**Authors:** MariNieves Pardo-Rodriguez, Erik Bojorges-Valdez, Oscar Arias-Carrion, Oscar Yanez-Suarez

**Affiliations:** ^1^Engineering Studies for Innovation, Universidad Iberoamericana Ciudad de México, Mexico City, Mexico; ^2^División de Neurociencias Clínica, Instituto Nacional de Rehabilitación Luis Guillermo Ibarra Ibarra, Mexico City, Mexico; ^3^Neuroimage Research Laboratory, Universidad Autónoma Metropolitana - Iztapalapa, Mexico City, Mexico

**Keywords:** neuromodulation, autonomic regulation, entrainment, heart rate variability (HRV), EEG-band power time series, breathing, Granger-causal relationship

## Abstract

**Introduction:**

The mechanisms by which conscious breathing influences brain-body signaling remain largely unexplored. Understanding how controlled breathing modulates neural and autonomic activity can offer insights into self-regulation and adaptive physiological control. This study investigates how conscious breathing affects cortical-autonomic communication by analyzing bidirectional interactions between EEG band power time series (BPts), heart rate variability (HRV), and breathing signals.

**Methods:**

Data were collected from fifteen healthy subjects during three experimental conditions: a spontaneous breathing state (Rest) and two controlled breathing tasks (CBT 1 and CBT 2). EEG recordings were analyzed to compute BPts across the δ, θ, α, β, and γ frequency bands, while HRV and breathing signals were derived from ECG data. Cross-spectrum analysis and Granger causality tests were performed between HRV and BPts. To further investigate directional interactions, Granger-causal relationships were explored between components of the BPts extracted using empirical mode decomposition and the HRV and breathing signals.

**Results:**

Bidirectional Granger-causal relationships were found between neural and autonomic systems, emphasizing the dynamic interaction between the brain and body. Specific BPts components mediated neural-autonomic communication, with one component consistently aligning with the frequency of conscious breathing (~0.05 Hz) during the CBTs. Cross-spectral peaks at this frequency and its harmonics highlight the role of respiratory entrainment in optimizing neuro-autonomic synchronization. Frequency-specific mechanisms observed in both fast and slow components reflect the complex regulation of autonomic functions through cortical modulation. The most prominent causal effects were observed in the γ band, suggesting its pivotal role in dynamic autonomic regulation, potentially acting as a communication pathway between the brain and body.

**Discussion:**

Our results demonstrate that conscious breathing enhances bidirectional cortical-autonomic modulation through frequency-specific dynamic neural mechanisms. These findings support a closed-loop model of physiological regulation driven by neural-respiratory entrainment and suggest that respiration can serve as a top-down mechanism for autonomic control. By clarifying how conscious breathing shapes brain-body dynamics, this work lays the foundation for research on neural self-regulation and supports the development of non-pharmacological interventions for improving mental and physiological health.

## 1 Introduction

Extensive electroencephalography (EEG) research has highlighted that the brain operates with a hierarchical organization, where slower oscillations modulate the amplitude of faster ones, thereby facilitating communication between distant brain areas and enhancing cognitive flexibility in response to internal or external demands (Klimesch, 2013, 2018; Lakatos et al., 2005; Siegel et al., 2012). This modulation supports the redistribution of oscillatory activity across frequency bands, maintaining functional equilibrium and optimizing information processing.

More recently, the concept of neuronal entrainment *i.e*., the synchronization of brain oscillations with rhythmic inputs has emerged as a key factor in influencing the temporal dynamics of cortical activity. Entrainment enhances interregional communication, optimizes information flow, and supports adaptive responses to environmental changes (Lakatos et al., 2019). Studies in animal models (Tort et al., 2018; Zhong et al., 2017) and humans with epilepsy (Herrero et al., 2018; Zelano et al., 2016) using intracranial recordings have shown that respiratory activity can phase-lock with neural oscillations and modulate the amplitude of higher-frequency rhythms. Furthermore, coupling between breathing and neural rhythms has been shown to promote parasympathetic dominance, improving information processing while reducing stress (Benson et al., 1974; Noble and Hochman, 2019). This dynamic interplay between neural activity and the autonomic system suggests that consciously modulating breathing could enhance both neural communication and autonomic regulation.

The autonomic nervous system (ANS) plays a crucial role in maintaining bodily regulation, balancing excitation and inhibition of involuntary signals throughout the body and brain (Saper, 2002). A key measure of ANS activity, heart rate variability (HRV), reflects its capacity to adapt to changing internal and external conditions, with increased variability indicating more robust autonomic control. HRV has been linked to cognitive and emotional performance, and has proven valuable in exploring the dynamics of autonomic regulation (Faes et al., 2017; Klimesch, 2018; Noble and Hochman, 2019; Pardo-Rodriguez et al., 2021a; Zaccaro et al., 2018).

Breathing directly influences HRV, with specific respiratory patterns shaping autonomic responses and overall physiological balance (Critchley et al., 2015). HRV is associated with respiratory sinus arrhythmia (RSA), a phenomenon where heart rate reflects the phases of breathing, further demonstrating the interconnectedness of autonomic processes (Klimesch, 2018; Porges, 2007). Conscious control of breathing such as slow, deep, or rhythmic patterns can enhance parasympathetic activation, reduce stress, and increase HRV, promoting relaxation and improving psychophysiological states (Ashhad et al., 2022; Benson et al., 1974; Noble and Hochman, 2019; Weng et al., 2021). Notably, breathing at 0.1 Hz (six breaths per minute) induces resonance between respiratory and cardiovascular rhythms, optimizing autonomic function by reinforcing these physiological signals (Ashhad et al., 2022; Mather and Thayer, 2018; Noble and Hochman, 2019; Zaccaro et al., 2018). This process is increasingly understood to involve the brain-heart axis, a complex network of neural, mechanical, and biochemical pathways linking central and autonomic functions (Valenza et al., 2025). This resonance enhances synchronization between the cardiovascular and respiratory systems, promoting autonomic control (Ashhad et al., 2022; Carnevali et al., 2013; Noble and Hochman, 2019).

Despite the growing popularity of breath-focused interventions in clinical and wellness contexts, the mechanisms by which specific breathing patterns could influence both neural and autonomic systems remain poorly understood. Recent studies have begun exploring how conscious breathing, combined with interoceptive attention, may influence brain oscillations and support autonomic regulation (Critchley and Garfinkel, 2015; Herrero et al., 2018; Pardo-Rodriguez et al., 2021a; Porges, 2007). The present study seeks to address this gap by examining how conscious modulation of breathing influences EEG oscillations and autonomic regulation.

Building on the concept of neuronal entrainment proposed by Lakatos et al. (2019), we suggest the brain uses breathing rhythms as pacing signals to modulate its oscillatory activity. Voluntary changes in breathing may influence vagal afferents by coupling respiration, baroreceptor inputs and *O*_2_ and *CO*_2_ exchange, inducing phase shifts that align both EEG activity and HRV with the breathing cycle. This modulation could recruit cortical rhythms at multiple frequencies, harmonically aligned with breathing, thereby influencing cortical temporal dynamics. In line with Lakatos et al. (2005) hierarchical oscillation theory, we propose that low-frequency respiratory rhythms modulate the amplitude of higher-frequency neural oscillations, enhancing interregional cortical communication and supporting cognitive flexibility. We thus hypothesize that conscious modulation of breathing enhances brain-body communication, improving neural synchronization and autonomic regulation compared to spontaneous breathing.

## 2 Materials and methods

### 2.1 Data

**Figure 1:**
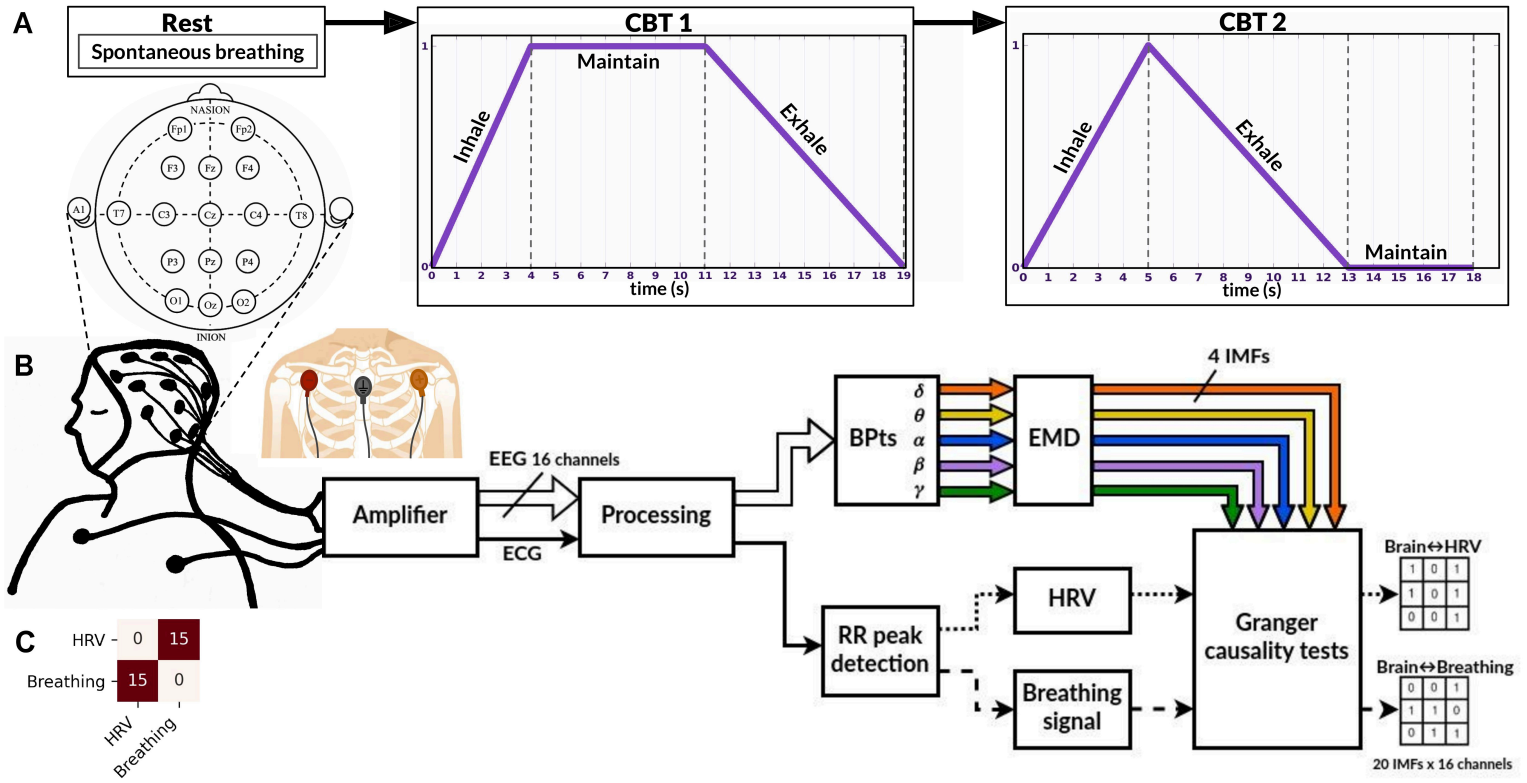
Experimental paradigm and processing pipeline. **(A)** Overview of the experimental paradigm, which includes three stages (conditions): **Rest**, **CBT 1** (controlled breathing task 1), and **CBT 2**. **(B)** Experimental setup and signal processing pipeline for each condition. Sixteen EEG channels were recorded to compute band power time series (*BPts*_*x*_), which were then decomposed using empirical mode decomposition (EMD) into four intrinsic mode functions (IMFs) per band and per channel. R-peaks were detected from the ECG signal and used to interpolate the HRV and breathing signals. Granger causality tests were performed on the resulting signals (see Methods for details). **(C)** Results of the Granger causality tests between the HRV and breathing signals for each condition. Across all 15 subjects, HRV G-caused breathing (first row, second column) and vice versa (second row, first column).

Data were collected from fifteen healthy subjects (seven female), aged 24 ± 3 years. Inclusion criteria were: normal body-mass index, no regular practice of an aerobic exercise, non-smoker, non-medicated, no known neuropathies or cardiovascular conditions, and no consumption of coffee or stimulating beverages two hours before the recordings. Subjects participated voluntarily after providing informed consent and received no monetary compensation. The experimental protocol followed the Declaration of Helsinki and had the approval of the University's Ethics Committee under record number 221. Recordings were scheduled between 10h 00 to 12h 00 to minimize any circadian influences on autonomic functions, as HRV can fluctuate throughout the day. Stimulus presentation and recording synchronization were done using BCI2000 software (Schalk et al., 2004). Signals were recorded using a g.USBamp (g.tec, Austria) amplifier at 1,200 samples per second, with a 60 Hz notch filter activated and the g.SCARABEO (g.tec, Austria) active electrode system. EEG signals were recorded from 16 channels placed according to the standard 10-20 system: *Fp1, Fp2, F3, Fz, F4, C3, Cz, C4, T7, T8, P3, Pz, P4, O1, O2*, and *Oz*. The reference used was *A1* and ground at *Fpz*. ECG signals were recorded by placing a pair of electrodes on the chest just below the left and right clavicles, and ground over the manubrium of the sternum (Figure 1B). Although this setup is not a clinical standard for ECG recordings, it was sufficient for detecting R-peaks, which are necessary for HRV estimation. Subjects were comfortably seated in a recliner armchair with head support from the moment they entered the recording area. Electrode setup took approximately 20 min, allowing for the transient phase of adaptation to pass and ensuring that participants' autonomic tone was at a relatively stable baseline before recordings began. These experiments were conducted at the Center for Well-Being (CBU, Centro del Bienestar Universitario) of Universidad Iberoamericana Ciudad de Mexico.

Although our sample size (*n* = 15) is relatively small, the consistency of directional interactions across nearly all participants suggests robust effects. Comparable studies in brain-heart dynamics have reported similar results with similar sample sizes (De la Cruz-Armienta et al., 2017; Faes et al., 2017; Pardo-Rodriguez et al., 2019; Umeno et al., 2002; Valenza et al., 2016). Nonetheless, expanding the participant pool in future research is essential to capture a broader variability in physiological responses and to better identify potential outliers. This would strengthen the generalizability of the observed effects and support more robust statistical inference.

### 2.2 Experimental design

The experimental protocol consisted of three conditions, depicted on Figure 1A, which were applied uniformly across all subjects. This design allowed us to compare spontaneous and consciously regulated breathing states to investigate whether specific breathing patterns enhance neuralautonomic coupling, as hypothesized. During all conditions, subjects remained in the same seated position with their eyes closed.

- **Resting condition (Rest):** Subjects were instructed to listen to an audio recording of “The Origin of Evil” by Leon Tolstoi, which lasted 6 min and 53 s (audio available at: The Origin of Evil). No instructions regarding breath control were given, therefore subjects engaged in spontaneous breathing during this phase.
- **CBT training 1:** Subjects were instructed and demonstrated how to perform a controlled breathing task (CBT), guided by an auditory cue (audios available at: CBTs training). The task was performed exclusively using nasal breathing. The cue indicated inhalation with an increasing frequency tone, maintenance by keeping a constant tone, and exhalation with a decreasing frequency tone. Data was recorded during this phase, but was not included in the analysis. For this training, subjects practiced a short, two-minute version of the CBT described below.
- **Controlled breathing task 1 (CBT 1):** Once subjects were able to synchronize their breathing with the auditory cue, they were instructed to perform **CBT 1**. This task consisted of breathing cycles with 4 seconds of inhalation, 7 s of breath-holding, and 8 s of exhalation. The task lasted 10 min, during which 32 cycles were performed.
- **CBT training 2:** The second controlled breathing training session was conducted in the same manner as described above for the subsequent and final condition. The task was performed exclusively using nasal breathing. Data was recorded during this phase, but was not included in the analysis. Once again, subjects practiced a short, two-minute version of the new CBT described below.
- **Controlled breathing task 2 (CBT 2):** CBT 2 consisted of breathing cycles with 5 s of inhalation, 8 s of exhalation, and 5 s of breath-holding. This task also lasted 10 min, with 34 cycles performed.

These breathing protocols were selected to approximate a lower harmonic of the well-studied 0.1 Hz resonance frequency (i.e., ~0.05 Hz), which has been associated with enhanced parasympathetic activation and autonomic regulation (Russo et al., 2017; Zaccaro et al., 2018). The 4-7-8 pattern is a well-established pranayama-based technique with documented effects on HRV, blood pressure, and stress reduction (Vierra et al., 2022). The 5-8-5 pattern, while less common in clinical studies, was designed to maintain a comparable cycle duration while modifying the phase distribution, consistent with slow-breathing principles used in respiratory training (Bhargava et al., 1988).

Although no direct respiratory signal (e.g., nasal thermistor) was recorded, participants were instructed to breathe exclusively through the nose. This was continuously monitored by the experimenter throughout the sessions. While slow, rhythmic breathing can be achieved via either the mouth or nose, nasal airflow has distinct neural effects that oral breathing lacks. Specifically, it has been shown to entrain cortical and limbic oscillations, via olfactory pathways, linked to the modulation of emotional states (Herrero et al., 2018; Pfurtscheller et al., 2025; Tort et al., 2025; Zelano et al., 2016).

### 2.3 Signal processing

**Figure 2:**
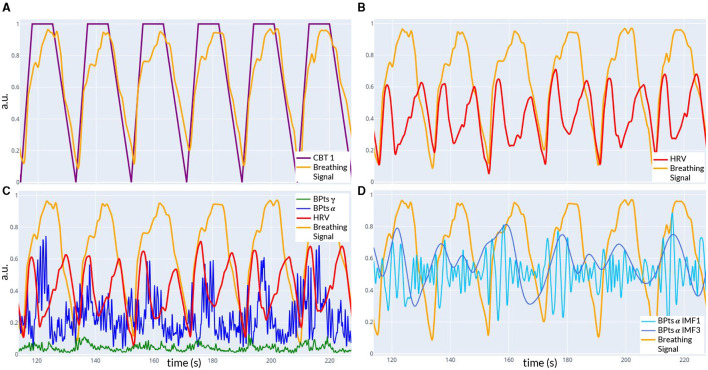
Example of signals recorded during **CBT 1**. All panels show data from the same time window of one trial. **(A)** Controlled breathing task (**CBT 1**, purple) that participants were instructed to follow, along with the breathing signal (light orange) recorded from one subject. The Pearson correlation coefficient between these two signals was 0.86. **(B)** Breathing signal (light orange) and HRV signal (red) from the same subject. **(C)** The signals from **(B)**, along with the band power time series *BPts*_α_ (blue) and *BPts*_γ_ (green) from the Cz channel. **(D)** Breathing signal (light orange), IMF 1 (light blue), and IMF 3 (dark blue) of *BPts*_α_ from **(C)**. The Pearson correlation coefficient between the breathing signal and IMF 3 was 0.21.

Figure 1B shows the experimental setup and the processing pipeline applied to each condition. Figure 2 provides an example of the signals recorded from one trial. All signals were filtered using a digital band-pass filter with cutoffs at 0.01 Hz and 100 Hz, consisting of a fourth-order Butterworth high-pass filter and an eighth-order Chebyshev II low-pass filter.

ECG signals were differentially obtained from the chest electrodes, and R-peaks of the QRS complex were identified using Hamilton's algorithm (Hamilton, 2002). HRV was determined from the time differences between these peaks and interpolated to a standard sampling rate of 10 samples per second using cubic interpolation. The breathing signal, derived from the amplitude of the R-peaks, was also interpolated to the same sampling rate using cubic interpolation.

Relative band power time series (BPts) for the five classical frequency bands (δ (1-4 Hz), θ (4-8 Hz), α (8-12 Hz), β (12-30 Hz), and γ (30-100 Hz)) were estimated for all EEG channels using a sliding window of two seconds with 95% overlap, resulting in a sampling rate of 10 samples per second. The Welch periodogram method was applied to each window, with internal parameters configured to assess a resolution of 0.5 Hz using a half-size window and 95% overlap. Relative power estimates were calculated by integrating the power spectral density (PSD) within each frequency band and normalizing by the total power (Figure 2C). This methodology has been previously described in the work of Pardo-Rodriguez et al. (2021a,b,c). All signals (BPts, HRV and breathing) were resampled for synchronization over the same temporal axis.

### 2.4 Analyses

#### 2.4.1 Band power analysis

To assess whether significant changes in band power occurred during the **CBTs**, between **Rest** vs **CBT1** and **Rest** vs **CBT2**, Shapiro-Walk normality tests were conducted on the data from all subjects, for each band and channel. For each pair of groups (different condition, same band and channel) with normal distributions, a parametric two-tailed t-test was performed. If normality was rejected for one of the groups, a Wilcoxon signed-rank test was used instead. The significance level for all statistical tests was set at 0.025, following a Bonferroni correction to account for multiple comparisons across the different conditions.

#### 2.4.2 Cross-spectrum analysis

Subsequently, the cross-spectrum between the normalized BPts and HRV signals was computed, yielding a spectral resolution of 0.01 Hz. For each frequency band and EEG channel, the amplitude at 0.05 Hz was compared between **CBT 1** and **Rest**, and the amplitude at 0.06 Hz was compared between **CBT 2** and **Rest**. These frequencies were chosen as they were the closest estimated frequencies to the breathing rate of each **CBT**. For each condition, band, and channel, normality was assessed using the Shapiro-Walk test. Depending on the outcome, either a paired t-test or a Wilcoxon signed-rank test was applied. Effect sizes were then calculated using Cohens d, based on all data from each **CBT** compared to **Rest**, along with the corresponding confidence intervals (CIs) and *p*-values. Values of Cohens d around 0.2 are generally considered small, around 0.5 medium, and 0.8 or higher large, indicating the strength of observed differences between mean cross-spectrum values across conditions.

#### 2.4.3 Empirical mode decomposition (EMD)

Following this, to analyze changes in terms of specific spectral components, four intrinsic mode functions (IMFs) were extracted from the BPts for each channel and frequency band using empirical mode decomposition (EMD) (Figure 2D). The EMD method (Huang Norden et al., 1998) was chosen for analyzing the BPts because it is particularly suited for non-stationary and non-linear processes, like BPts. The Python EMD package was used to compute the decomposition, specifically employing the *emd*.*sift*.*sift* function with default parameters (Quinn et al., 2021). Unlike traditional methods such as the STFT (short-time Fourier transform) or filter banks, which assume stationarity, EMD adapts to the data, extracting IMFs that reflect dynamic, time-varying oscillations without predefined frequency limits. This makes EMD ideal for capturing the complex temporal characteristics of the BPts.

Although EMD can introduce artifacts such as mode mixing, we ensured the reliability of the IMFs chosen by assessing the power spectral density (PSD) of each IMF and verifying the consistency of their bandwidths across subjects. The dominant frequency content of each IMF, identified as the peak frequency and the range exceeding 90% of peak power was calculated across all subjects, channels and bands, separately for each condition. The resulting frequencies were highly stable across tasks, with averaged values as follows: IMF 1 peaked at 0.262 Hz (range: 0.246–0.277 Hz), IMF 2 at 0.136 Hz (0.130–0.142 Hz), IMF 3 at 0.059 Hz (0.057–0.062 Hz), and IMF 4 at 0.026 Hz (0.025–0.027 Hz). Notably, the dominant frequency peaks for each IMF were distinct and showed no overlap.

#### 2.4.4 Causal analysis

Granger causality tests were first performed between each BPts (per channel) and the HRV signal to capture directional dependencies that may reflect neural-autonomic coupling. Granger causality is a statistical method used to determine whether past values of one time series can help predict future values of another time series (Granger, 1969). The test compares models that predict a signal using its own past values against models that include past values of another signal. If incorporating the second signal improves prediction accuracy significantly (*p* < 0.01), the first signal is said to G-cause the second. However, this causal relationship does not imply a direct physical connection between the signals, just a predictive dependency that underscores some form of functional connectivity. Effect sizes were then calculated using the Odds Ratio (OR), based on all data from each **CBT** compared to **Rest**, along with the corresponding CIs and *p*-values. OR values above 1 indicate increased likelihood of positive G-causal relationships during the **CBTs** compared to **Rest**, whereas values below 1 suggest a decreased likelihood.

Finally, Granger causality tests were also performed between each IMF (by band and channel) and the HRV and breathing signals.

Recent studies have highlighted Partial Directed Coherence (PDC) as a frequency-domain alternative for assessing directional connectivity in EEG, particularly in relation to HRV and event-related desynchronization (Al-Ezzi et al., 2024; Molloy et al., 2023). While Granger causality operates in the time domain and is well-suited for examining short, transient neural-autonomic dynamics, future work could integrate PDC to explore band-specific or long-range neural-autonomic interactions more precisely.

## 3 Results

### 3.1 Band power analysis

Mean BPts increased in the δ, β, and γ bands during both **CBTs** across nearly all channels when compared to **Rest**, as shown in Tables 1, 2. However, these differences were only significant for *BPts*_β_ at P3 and Pz for both **CBTs**; and for *BPts*_γ_ at Pz for both **CBTs**, and at Cz, T7, P3, P4, O1 and O2, for **CBT 1**. In contrast, for **CBT 1**, significant decreases in mean BPts were observed in the θ and α bands from central through occipital channels, excluding Pz. For **CBT 2**, significant decreases were found in *BPts*_θ_ at T7 and in *BPts*_α_ at P3, P4, Oz, O1 and O2.

**Table 1:** Mean relative BPts differences between Rest and CBT 1 in arbitrary units.

**Channel**	**δ**	**θ**	**α**	**β**	**γ**		**δ**	**θ**	**α**	**β**	**γ**
Fp1	0.026	−0.005	−0.021	−0.000	0.004	Fp2	0.015	−0.009	−0.026	−0.002	0.028
F3	0.026	−0.013	−0.019	−0.000	0.012	F4	0.028	−0.012	−0.025	0.000	0.013
Fz	0.018	−0.010	−0.018	0.001	0.014	Cz	0.010	−0.017^**^	−0.013	0.007	0.017^*^
C3	0.022	−0.016^**^	−0.019^*^	0.005	0.014	C4	0.028	−0.020^**^	−0.019^**^	0.001	0.015
T7	0.000	−0.014^**^	−0.019^*^	0.008	0.029^**^	T8	0.026	−0.021^**^	−0.031^**^	0.004	0.029
P3	0.016	−0.012^**^	−0.030^**^	0.011^*^	0.018^**^	P4	0.019	−0.015^**^	−0.030^**^	0.009	0.020^**^
Pz	−0.003	−0.010	−0.020	0.015^**^	0.019^**^	Oz	0.016	−0.014^*^	−0.051^**^	0.004	0.051
O1	0.024	−0.017^**^	−0.053^**^	0.004	0.048^**^	O2	0.033	−0.017^**^	−0.064^**^	0.004	0.050^**^

Comparisons were made across the respective frequency band and channel for all subjects, between the two conditions (**Rest** and **CBT 1**) using either a two-tailed t-test or a Wilcoxon signed-rank test, depending on the normality of the data. Significant differences are indicated by ^*^ and ^**^ next to each difference, where *p* < 0.025 and *p* < 0.01, respectively. Positive values indicate an increase during **CBT 1**.

**Table 2:** Mean relative BPts differences between Rest and CBT 2 in arbitrary units.

**Channel**	**δ**	**θ**	**α**	**β**	**γ**		**δ**	**θ**	**α**	**β**	**γ**
Fp1	0.022	0.001	−0.022	−0.002	0.004	Fp2	0.006	0.006	−0.011	0.015	−0.016
F3	0.032	−0.005	−0.024	−0.002	0.003	F4	0.026	−0.002	−0.026	0.000	0.004
Fz	0.011	−0.000	−0.013	0.001	0.004	Cz	0.004	−0.008	−0.008	0.005	0.009
C3	0.019	−0.007	−0.018	0.003	0.004	C4	0.004	−0.006	−0.009	0.005	0.007
T7	0.020	−0.009^**^	−0.019	−0.001	0.011	T8	0.008	−0.009	−0.030	0.007	0.029
P3	0.027	−0.004	−0.038^*^	0.005^*^	0.009	P4	0.015	−0.003	−0.030^*^	0.007	0.012
Pz	−0.005	−0.001	−0.026	0.010^*^	0.012^*^	Oz	0.032	−0.002	−0.051^*^	0.001	0.034
O1	0.036	−0.005	−0.060^*^	0.001	0.032	O2	0.039	−0.004	−0.068^**^	0.002	0.034

Comparisons were made across the respective frequency band and channel for all subjects, between the two conditions (**Rest** and **CBT 2**) using either a two-tailed t-test or a Wilcoxon signed-rank test, depending on the normality of the data. Significant differences are indicated by ^*^ and ^**^ next to each difference, where *p* < 0.025 and *p* < 0.01, respectively. Positive values indicate an increase during **CBT 2**.

### 3.2 Cross-spectrum analysis

**Figure 3:**
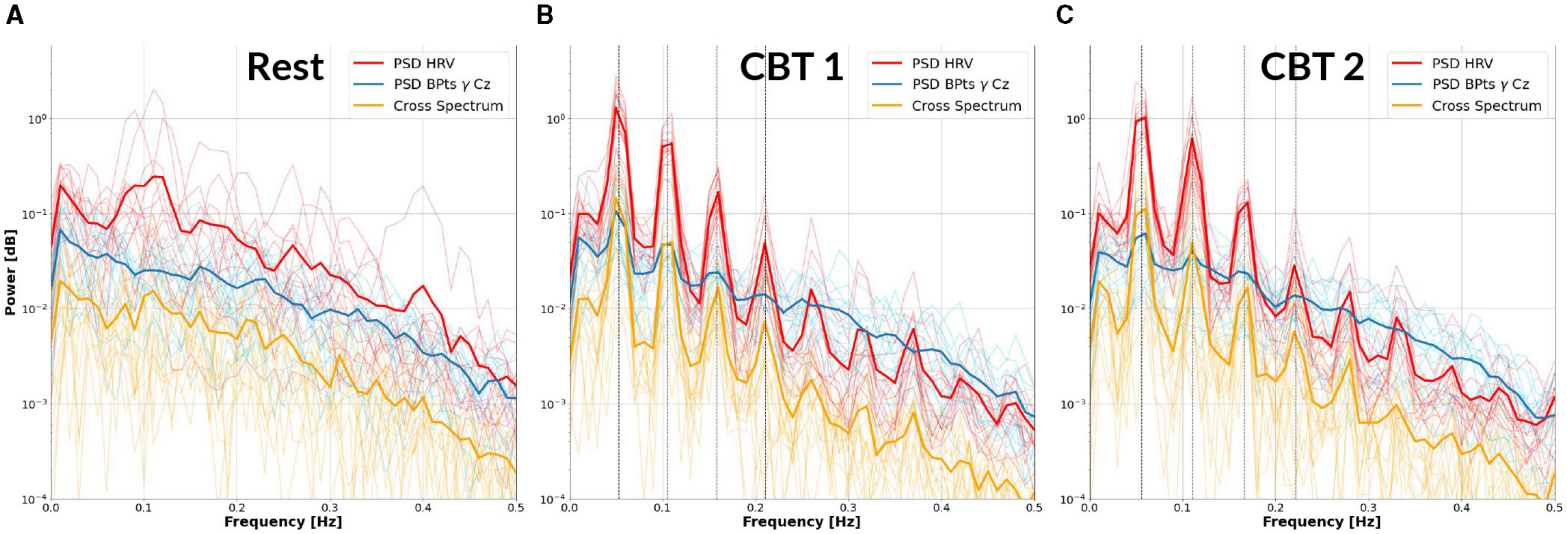
Cross-Spectrum between HRV and *BPts*_γ_ at Cz. Each panel displays the mean power spectral density (PSD) of the HRV (red) and *BPts*_γ_ at Cz (blue), as well as their cross-spectrum (yellow), averaged across subjects (bold lines). Individual subject traces are shown as fine lines. **(A)** Results for **Rest**. **(B)** Results for **CBT 1**. Vertical lines mark the frequency of **CBT 1** (19-second breathing cycles, i.e., 0.0526 Hz) and its following three harmonics. **(C)** Results for **CBT 2**, with vertical lines marking the frequency of **CBT 2** (18-second breathing cycles, i.e., 0.0555 Hz) and its following three harmonics. In both **(B, C)**, all signals show peak power at the respective fundamental frequency, followed by progressively smaller peaks at subsequent harmonics.

Figure 3 provides initial evidence of neural and autonomic coupling driven by controlled breathing. The cross-spectrum quantifies the degree to which two signals share spectral power at specific frequencies, reflecting their frequency-domain coherence. In Figures 3B, C, the dominant spectral peak is centered at the respective **CBT** frequency, followed by progressively smaller peaks at its harmonics indicating a cyclical interaction likely entrained by the breathing pattern. These harmonics are consistent with the periodic structure of the controlled breathing tasks and suggest coherent phase-locking between neural activity and cardiorespiratory rhythms. In contrast, Figure 3A shows that during **Rest**, the cross-spectrum is more broadly distributed across frequencies, with no prominent peaks, suggesting the absence of such coupling.

**Figure 4:**
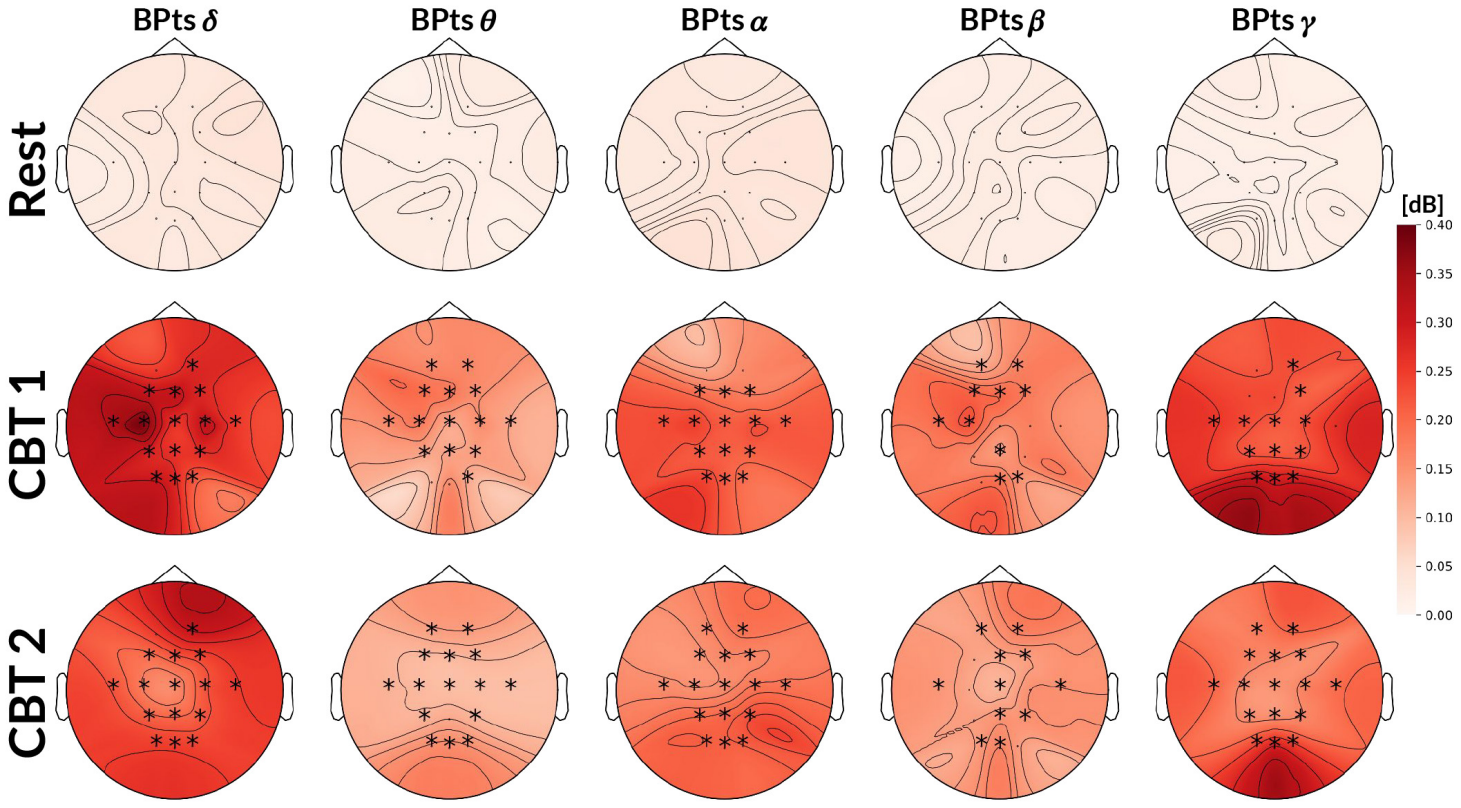
Topographic cross-spectrum values between HRV and BPts. Each topographic map shows the mean cross-spectrum value [dB] between the normalized HRV and corresponding BPts at each channel, averaged across subjects. The first row shows results for **Rest** at 0.05 Hz, the second for **CBT 1** at 0.05 Hz, and the third for **CBT 2** at 0.06Hz. Statistical comparisons were performed between **Rest** and each **CBT** at the same frequency (i.e., 0.05 Hz for **CBT 1** and **Rest**, 0.06 Hz for **CBT 2** and **Rest**), using either a two-tailed t-test or a Wilcoxon signed-rank test depending on normality. Channels with significant differences (*p* < 0.001) are marked with a *.

Figure 4 shows that cross-spectrum values between HRV and BPts during the **CBTs**, at the frequencies closest to the respective breathing rates (*i.e*., 0.05 Hz for **CBT 1** and 0.06 Hz for **CBT 2**), increased significantly across subjects compared to **Rest**. Effect sizes measured through Cohen's d were 1.37 (95% CI: [1.27, 1.47]; *p* < 0.001) for **CBT 1** vs. **Rest**, and 1.45 (95% CI: [1.34, 1.55]; *p* < 0.001) for **CBT 2** vs **Rest**. This means that controlled breathing was strongly associated with enhanced frequency-specific coupling between neural and autonomic signals.

### 3.3 Granger causality

#### 3.3.1 BPts → HRV

**Figure 5:**
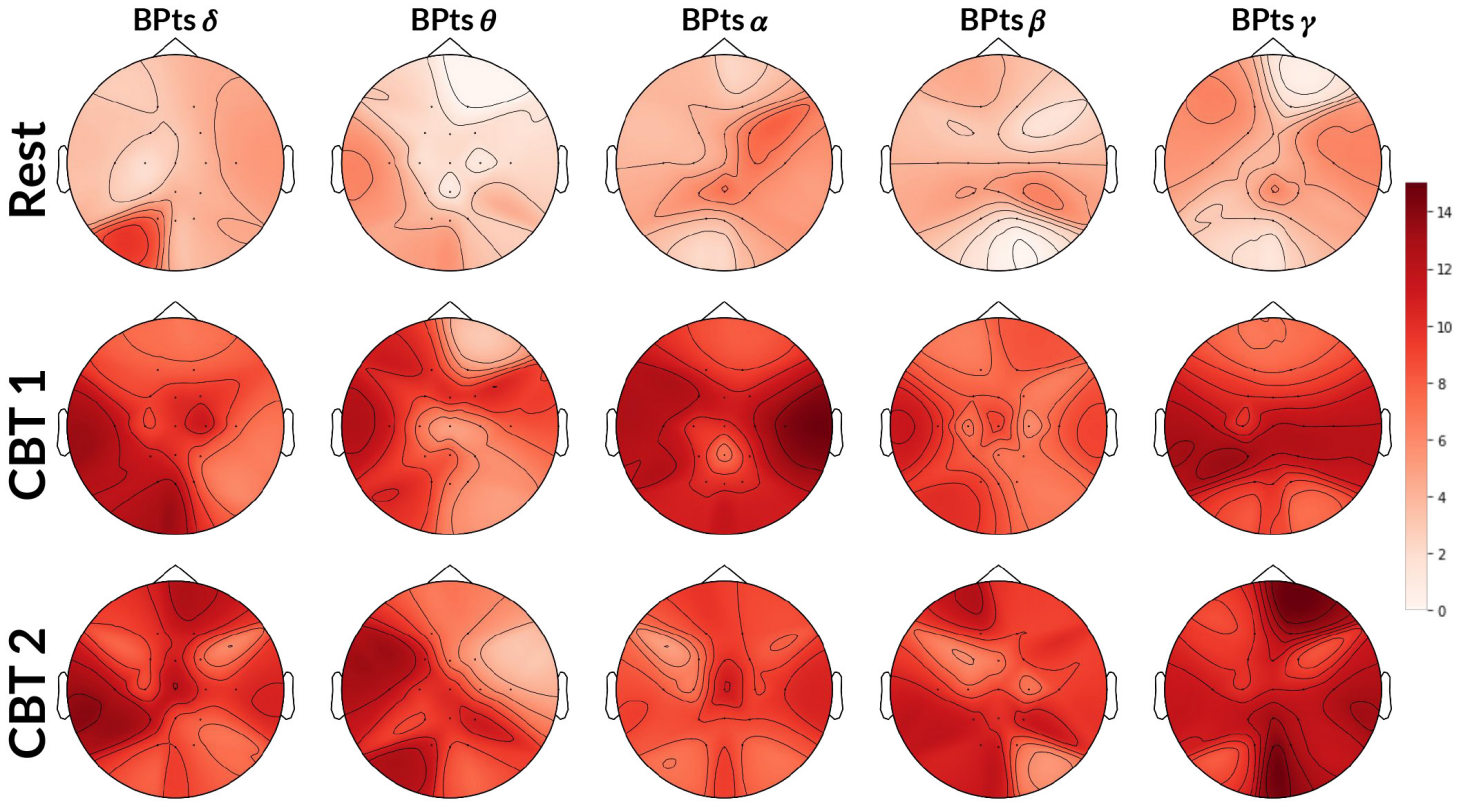
Topographic BPts → HRV Granger causality tests. Each topographic map shows the number of subjects (maximum 15) exhibiting positive G-causal relationships from each BPts per channel to the HRV signal. The first row shows results for **Rest**, the second for **CBT 1**, and the third for **CBT 2**. Compared to **Rest**, the number of subjects with positive G-causal relationships increased across all frequency bands during both **CBT 1** and **CBT 2**.

Topographic maps in Figure 5 show an increase in G-causal relationships from BPts → HRV across all frequency bands during both **CBTs** compared to **Rest**. The γ band exhibited the highest number of subjects with positive G-causality overall, while the θ band showed the greatest increase relative to **Rest**. Effect size analysis indicated that subjects were 4.74 times more likely to show positive G-causality during **CBT 1** compared to **Rest** (*OR* = 4.74; 95% CI: [3.89, 5.79]; *p* < 0.001), and 5.13 times more likely during **CBT 2** (*OR* = 5.13; 95% CI: [4.20, 6.27]; *p* < 0.001).

#### 3.3.2 HRV → BPts

**Figure 6:**
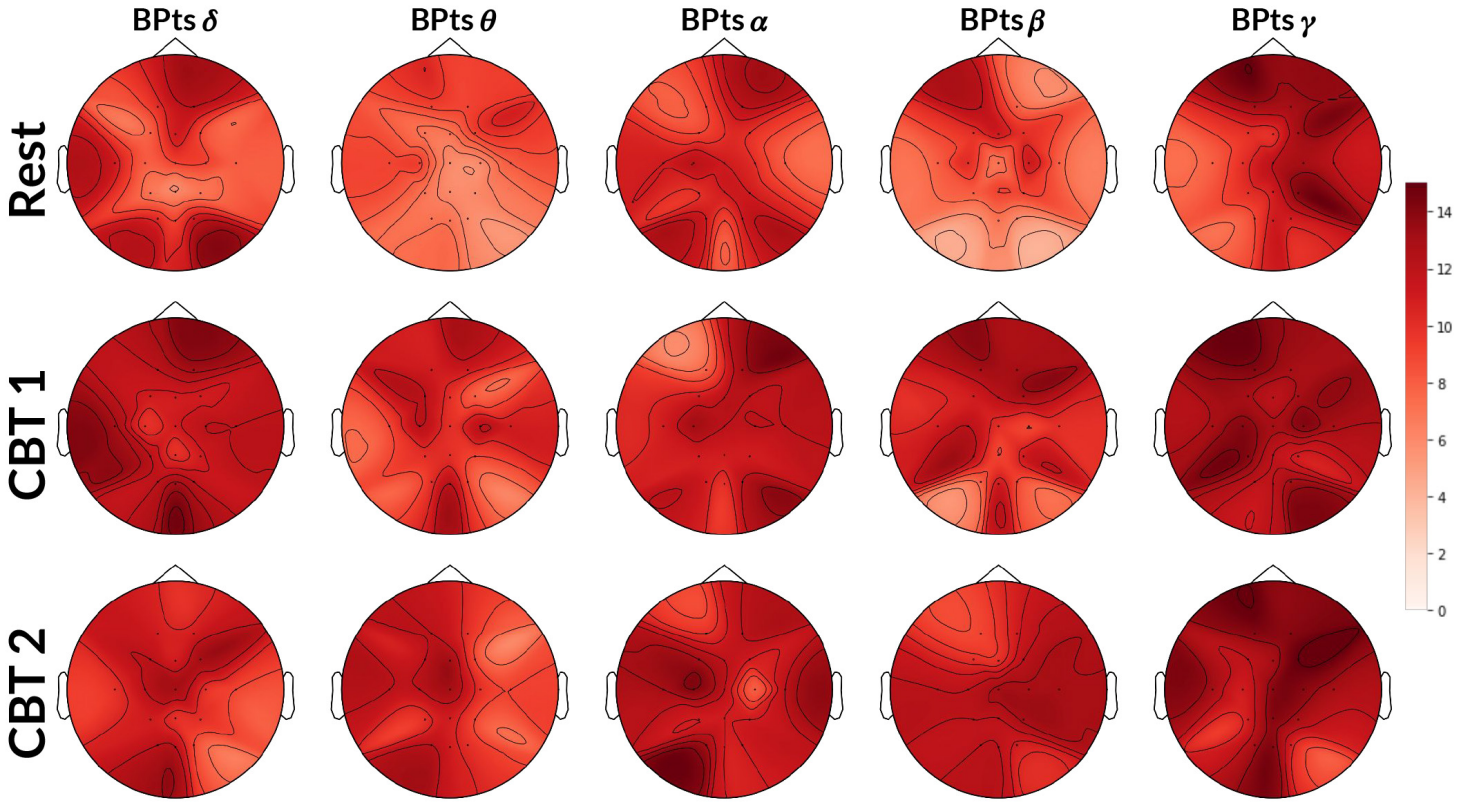
Topographic HRV → BPts Granger causality tests. Each topographic map shows the number of subjects (maximum 15) exhibiting positive G-causal relationships from the HRV signal to each BPts per channel. The first row shows results for **Rest**, the second for **CBT 1**, and the third for **CBT 2**. Compared to **Rest**, the number of subjects with positive G-causal relationships increased across all frequency bands during both **CBT 1** and **CBT 2**.

Topographic maps in Figure 6 show a higher total number of G-causal relationships from HRV → BPts during both **CBTs**, with increases observed across all frequency bands compared to **Rest**. Again, the γ band showed the highest number of subjects with positive G-causality overall, while the θ band showed the greatest relative increase. However, unlike the BPts → HRV direction, HRV → BPts already exhibited a higher baseline level of G-causality during **Rest**. As a result, the observed increases during the **CBTs**, though more numerous in absolute terms, were smaller in relative magnitude. Effect size analysis showed that subjects were 1.93 times more likely to exhibit positive G-causality during **CBT 1** compared to **Rest** (*OR* = 1.93; 95% CI: [1.58, 2.36]; *p* < 0.001), and 2.10 times more likely during **CBT 2** (*OR* = 2.10; 95% CI: [1.71, 2.58]; *p* < 0.001).

**Figure 7:**
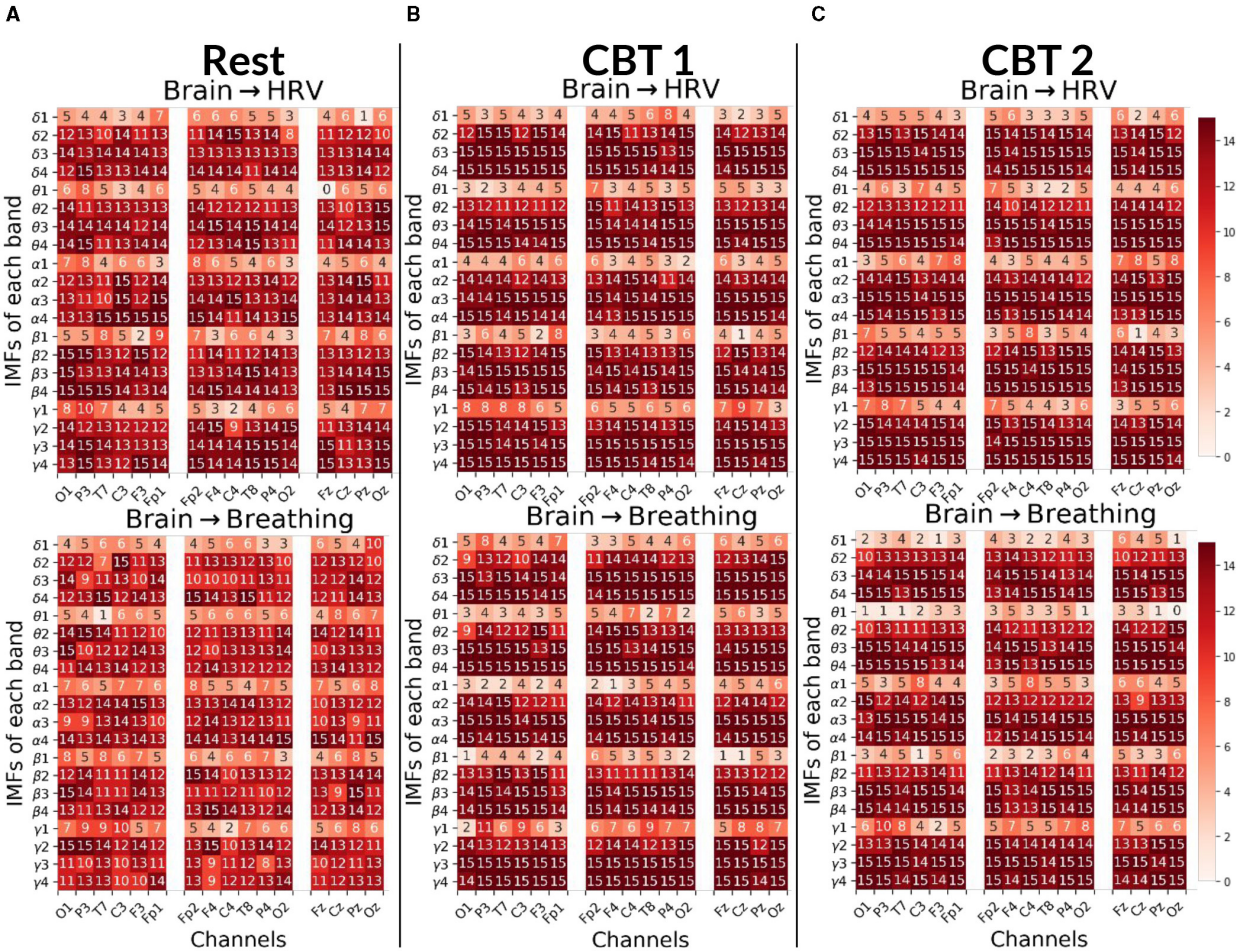
Brain → Body Granger causality tests. Each panel displays the number of subjects (maximum 15) with positive G-causal relationships from each intrinsic mode function (IMF), per frequency band and channel, to either the HRV (top) or breathing (bottom) signal. In the first column, channels are ordered from occipital to frontal along the left hemisphere; in the second column, from frontal to occipital along the right hemisphere; and in the third column, from frontal to occipital along the midline. **(A)** Results for **Rest**. **(B)** Results for **CBT 1**. **(C)** Results for **CBT 2**. Compared to **Rest**, both **CBT 1** and **CBT 2** showed increased G-causal relationships for IMFs 3 and 4 across all frequency bands.

**Figure 8:**
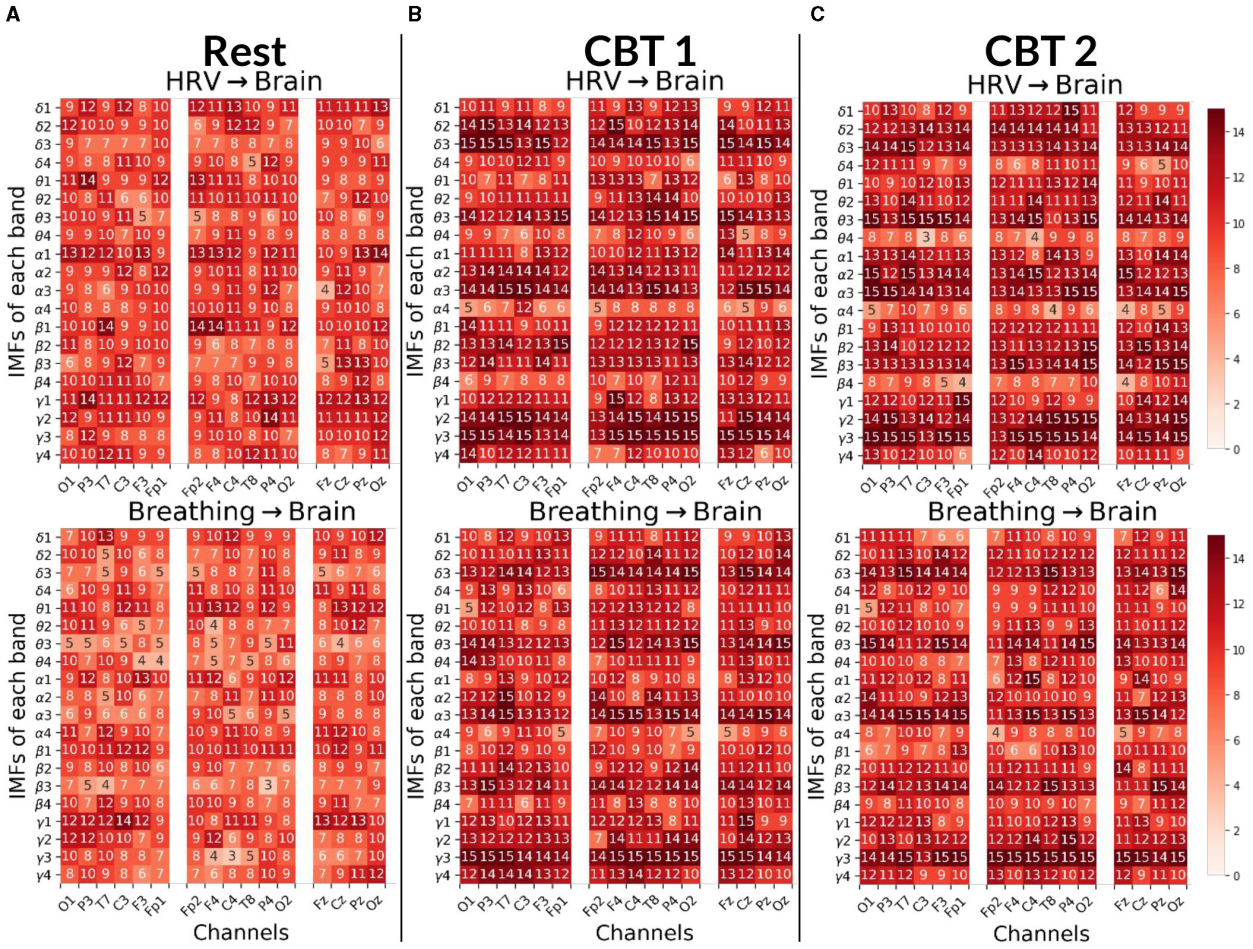
Body → Brain Granger causality tests. Each panels displays the number of subjects (maximum 15) with positive G-causal relationships from either the HRV (top) or breathing (bottom) signal to each intrinsic mode function (IMF) per frequency band and channel. In the first column, channels are ordered from occipital to frontal along the left hemisphere; in the second column, from frontal to occipital along the right hemisphere; and in the third column, from frontal to occipital along the midline. **(A)** Results for **Rest**. **(B)** Results for **CBT 1**. **(C)** Results for **CBT 2**. Compared to **Rest**, both **CBT 1** and **CBT 2** showed increased G-causal relationships for IMFs 2 and 3 across all frequency bands.

To gain finer insight into the spectral components of the BPts and their relationship with HRV and respiration, we next applied Granger causality analyses to the BPts decomposed via EMD. For this decomposition analysis, we adopt the terms **brain → body** and **body → brain** to reflect the broader conceptual framing, where “brain” refers to the IMFs of BPts, and “body” encompasses both HRV and breathing signals. Figure 7 shows results for the **brain → body** direction, while Figure 8 shows the results for the **body → brain** direction.

#### 3.3.3 Brain → body

Figure 7 shows that, for both **CBTs**, G-causal relationships increased for IMF 3 and IMF 4 across all frequency bands compared to **Rest**. The greatest increase was observed for **brain → breathing** relationships on IMF 3, particularly in the δ, α and γ bands, and on IMF 4 in the γ band. Additionally, **brain → HRV** relationships increased for IMF 2 in δ, α and γ bands. In contrast, G-causal relationships for IMF 1 decreased across most frequency bands, except for γ during **CBT 1**, in the majority of channels. Channel distribution shows that G-causal relationships increased predominantly over central and frontal channels for **brain → HRV** in the β and γ bands, and for **brain → breathing** in the γ band, as well as for IMF3 across all bands except α, during both **CBTs** compared to **Rest**.

#### 3.3.4 Body → brain

Figure 8 shows that, for both **CBTs**, G-causal relationships increased for IMFs 2 and 3 across all frequency bands compared to **Rest**; with the greatest increase observed for **breathing → brain** relationships on IMF 3. Additionally, **breathing → brain** relationships increased on IMF 4 in the θ and γ bands; and, in the majority of channels, for **HRV → brain** on IMF 4 in γ. In contrast, **HRV → brain** relationships decreased on IMF 4 in the θ, α and β bands, and on IMF 1 in the δ band during **CBT 1**, and the γ band during **CBT 2**. **Breathing → brain** relationships also decreased for IMF 1 in the θ and β bands, as well as in δ band during **CBT 2** and the α band during **CBT 1**.

Interestingly, channel distribution shows that G-causal relationships increased predominantly over central and frontal channels for **HRV → brain** on IMF3 in the θ and γ bands, and for **breathing → brain** in the δ and γ bands during both **CBTs** compared to **Rest**. However, G-causal relationships increased over temporal, parietal and occipital channels, for **HRV → brain** for IMF3 in the δ and α bands, and for **breathing → brain** in the β and α bands during both **CBTs** compared to **Rest**.

### 3.4 Spectral component analysis

**Figure 9:**
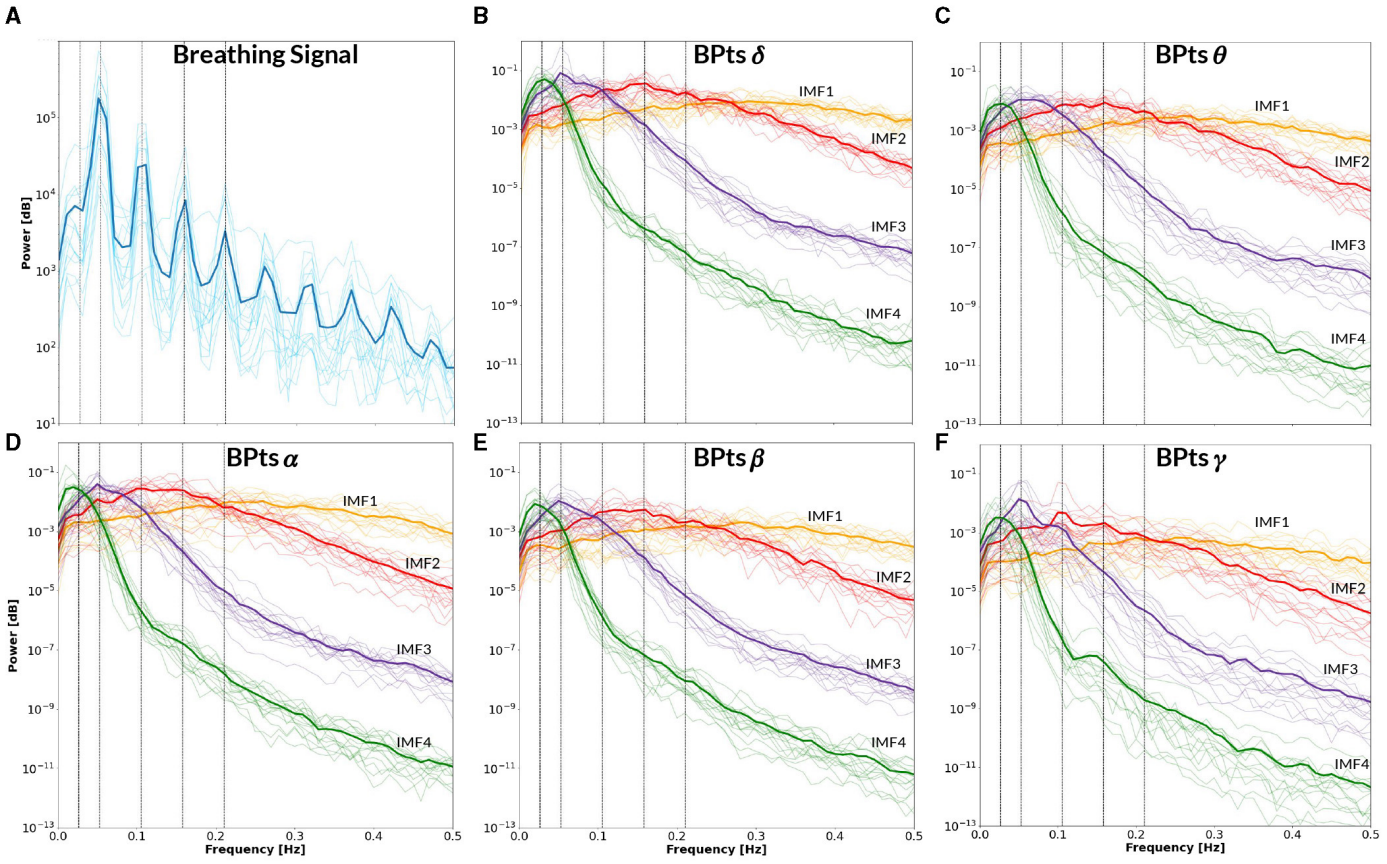
Power spectral density–**CBT 1**. All panels display the mean power spectral density (PSD) averaged across subjects (bold lines), with individual subject PSDs shown as finer lines. **(A)** PSD of the breathing signal (blue), where the highest peak occurs at the fundamental frequency, followed by smaller peaks at subsequent harmonics. Vertical lines indicate the frequency of **CBT 1** (19-second breathing cycles, *i.e*. 0.0526 Hz) and its half, second, third and fourth harmonics. **(B–F)** Show the PSD of the band power time series (*BPts*) for each frequency band and intrinsic mode function (IMF 1 in yellow, IMF 2 in red, IMF 3 in purple, IMF 4 in green). **(B)** PSD *BPts*_δ_. **(C)** PSD *BPts*_θ_. **(D)** PSD *BPts*_α_. **(E)** PSD *BPts*_β_. **(F)** PSD *BPts*_γ_. For all bands, IMF 4 centers around the half harmonic, while IMF 3 is centered at the fundamental frequency. Additionally, IMF 3 shows a prominent peak at the breathing frequency in all bands except θ, while IMF 2 shows a smaller peak at the second harmonic exclusively in γ. Notably, IMF 4 in the γ band also exhibits a secondary peak around ~0.15 Hz.

**Figure 10:**
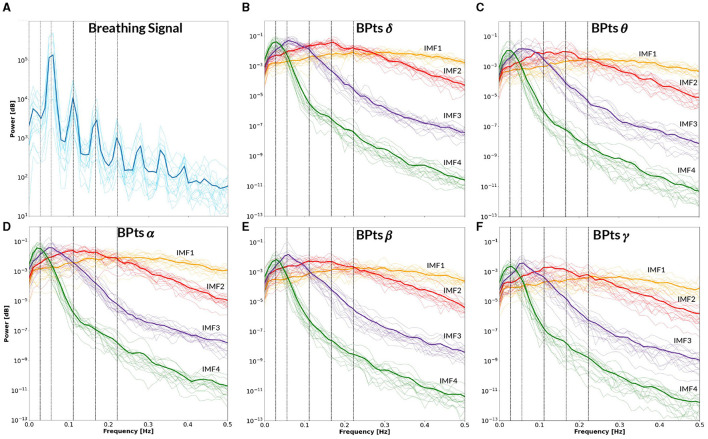
Power spectral density–**CBT 2**. All panels display the mean power spectral density (PSD) averaged across subjects (bold lines), with individual subject PSDs shown as finer lines. **(A)** PSD of the breathing signal (blue), where the highest peak occurs at the fundamental frequency, followed by smaller peaks at subsequent harmonics. Vertical lines indicate the frequency of **CBT 2** (18-second breathing cycles, *i.e*. 0.0555 Hz) and its half, second, third and fourth harmonics. **(B–F)** Show the PSD of the band power time series (*BPts*) for each frequency band and intrinsic mode function (IMF 1 in yellow, IMF 2 in red, IMF 3 in purple, IMF 4 in green). **(B)** PSD *BPts*_δ_. **(C)** PSD *BPts*_θ_. **(D)** PSD *BPts*_α_. **(E)** PSD *BPts*_β_. **(F)** PSD *BPts*_γ_. For all bands, IMF 4 centers around the half harmonic, while IMF 3 is centered at the fundamental frequency. Additionally, IMF 3 shows a prominent peak at the breathing frequency in all bands except θ.

**Figure 11:**
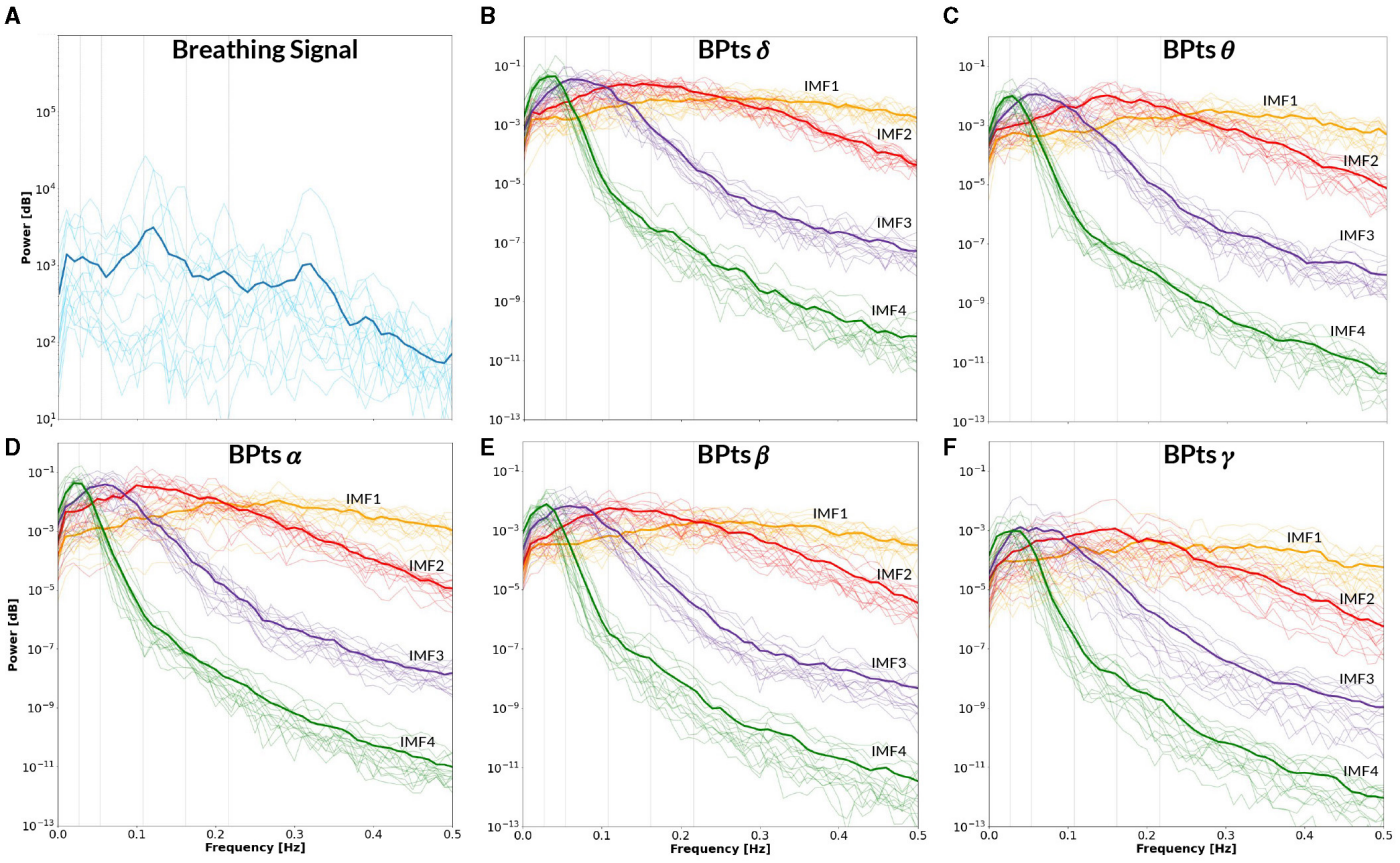
Power spectral density–**Rest**. All panels display the mean power spectral density (PSD) averaged across subjects (bold lines), with individual subject PSDs shown as finer lines. **(A)** PSD of the breathing signal during spontaneous breathing (blue). Two prominent peaks appear around ~0.1 Hz and ~0.32 Hz, consistent with spontaneous breathing rhythms. Vertical gray lines indicate the mean frequency between **CBT 1** and **CBT 2** (*i.e*. 0.054 Hz) and its half, second, third and fourth harmonics. **(B–F)** Show the PSD of the band power time series (*BPts*) for each frequency band and intrinsic mode function (IMF 1 in yellow, IMF 2 in red, IMF 3 in purple, IMF 4 in green). **(B)** PSD *BPts*_δ_. **(C)** PSD *BPts*_θ_. **(D)** PSD *BPts*_α_. **(E)** PSD *BPts*_β_. **(F)** PSD *BPts*_γ_.

Figure 9 presents the BPts spectral analysis results for **CBT 1**, Figure 10 for **CBT 2** and Figure 11 for **Rest**. These figures illustrate how, despite EMD being a non-linear decomposition method, each IMF demonstrated a consistent frequency range and content across subjects. Importantly, because EMD is a data-driven method, it does not rely on predefined frequency bands or filtering assumptions. This allows the extracted IMFs to reflect intrinsic oscillatory dynamics rather than imposed spectral boundaries. Furthermore, IMF 3 displayed a clear relationship with each **CBT** (Figures 9, 10), exhibiting a peak in all frequency bands, except for θ, centered at the respective **CBT** frequency.

For each IMF and frequency band from the Cz channel, a cross-correlation analysis was performed with each subject's breathing signal. The respective IMF was adjusted to the optimal lag, and the resulting Pearsons coefficient between the signals was calculated. The highest average correlation across subjects was observed for IMF 3 in the γ band during **CBT 2**, with a value of 0.307, indicating a weak correlation, which suggests a less direct or potentially artificial relationship with the physical phenomenon. Nonetheless, both signals shared a similar dominant frequency, suggesting that IMF 3 still captured oscillatory dynamics temporally aligned with the breathing rhythm, thereby supporting the notion of frequency-based coupling even in the absence of strong amplitude correlation.

Notably, Figure 9F reveals a secondary peak in IMF 4 centered at ~0.15 Hz, which was observed only in the γ band during **CBT 1**. According to Klimesch (2018) binary hierarchy model, this frequency (~0.16 Hz) represents one of three preferred breathing rates linked to different affective and cognitive states (Klimesch, 2018; Pfurtscheller et al., 2025). While the dominant frequency in **CBT 1** corresponds to the instructed breathing rhythm (~0.05 Hz), the emergence of a peak near 0.15 Hz may reflect an endogenous modulation mechanism operating within a harmonically related frequency range, as predicted by the binary hierarchy model.

The PSD of the breathing signal reveals a distinct cyclical phenomenon in both **CBTs** (Figures 9A, 10A), with the highest peak centered around the respective **CBT** frequency, followed by smaller peaks at subsequent harmonics. This pattern indicates that the controlled breathing exercise was effectively performed in both conditions. Furthermore, after adjusting each subject's breathing signal based on the optimal lag identified through cross-correlation with the respective **CBT**, the average Pearsons correlation coefficients across subjects were 0.82 for **CBT 1** and 0.81 for **CBT 2**. In this context, a high correlation could indicate both accurate performance of the CBT and adequate estimation of the breathing signal.

Furthermore, the PSD of the breathing signal during the **Rest** condition (Figure 11) showed spectral peaks centered around ~0.1 Hz and ~0.32 Hz. These spontaneous breathing rates align with previously reported patterns observed in both healthy participants and patients undergoing MRI sessions (Pfurtscheller et al., 2019, 2025; Zelano et al., 2016). The presence of a ~0.32 Hz peak is consistent with one of the preferred breathing frequencies proposed by the binary hierarchy model (Klimesch, 2018), suggesting that even in resting conditions, participants may exhibit spontaneous breathing rhythms with functional significance.

## 4 Discussion

The primary finding of this study is the observation of bidirectional G-causal relationships between brain BPts and autonomic events, specifically HRV and breathing, during **CBTs**. The decomposition of BPts into IMFs reveals distinct spectral components that are differentially modulated across conditions, suggesting that each IMF reflects varying physiological mechanisms underlying these interactions.

An important consideration when interpreting these results is ensuring the observed G-causal relationships are not experimental artifacts. One compelling argument against this is the consistent positive G-causal relationships observed between HRV and breathing signals across all subjects and conditions, as shown in Figure 1C. HRV is tightly coupled with breathing via RSA, where RR intervals decrease during inhalation and increase during exhalation (Klimesch, 2018). The bidirectional positive relationships in all subjects confirm their common origin. Additionally, to support the notion that the observed relationships are not artifactual, the low subject-level correlation values, specifically for IMF 1 in Figure 7 and IMF 4 in Figure 8, suggest that the **brain↔body** G-causal interactions are unlikely to reflect muscular artifacts in the EEG. Moreover, the highest observed cross-correlation between breathing and EEG components (IMF 3 in the γ band during **CBT 2**) was only 0.307, indicating a weak amplitude coupling. This further supports the idea that frequency alignment, rather than volume-conducted muscle activity, underlies the observed effects.

Further, the decomposition of BPts into IMFs, in conjunction with the observed G-causal relationships, suggests that **brain↔body** communication occurs through distinct spectral pathways. Each IMF appears to capture distinct aspects of the underlying physiological processes, with each spectral feature potentially representing a unique component of the communication between brain and body. This provides a more comprehensive picture of how these systems interact, highlighting the role of frequency-specific mechanisms in the bidirectional transfer of information (Mitra et al., 2016).

Notably, IMF 3 showed frequency alignment with the conscious breathing rate during both **CBT 1** and **CBT 2**, but not during **Rest**, suggesting entrainment of cortical activity to the respiratory rhythm (Figures 2D, 9, 10) (Tort et al., 2025). Cross-spectrum analyses further revealed increased spectral power at the breathing frequency and its harmonics, indicating frequency-specific coordination between neural and autonomic signals (Figures 3, 4). These patterns were not present during **Rest**, reinforcing the idea that conscious breathing provides a temporal reference for brain and autonomic synchronization (Klimesch, 2013, 2018; Mather and Thayer, 2018). Additionally, we observed increased bidirectional statistical dependencies (via Granger causality) between BPts and autonomic signals during the **CBTs**, particularly for IMF 3. While these dependencies do not imply direct physiological causation, they highlight dynamic, task-related interactions between cortical and autonomic systems; such dependencies may arise from shared influence by a third, unknown common driver, a possibility that remains an open question. The increase in the **body → brain** direction, particularly in the **HRV → brain** relationships on IMF 2 (Figure 8), suggests that faster BPts components may be modulated by autonomic signals like HRV. In contrast, IMF 4 showed an increase in the **brain → body** direction (Figure 7), suggesting that slower BPts components may reflect a more delayed, regulatory interaction between the brain and the heart. These findings align with previous work showing that respiratory rhythms can entrain brain activity in cortical and limbic regions (Zelano et al., 2016; Herrero et al., 2018), and support the broader idea that conscious breathing enhances communication between neural and autonomic systems.

These results are also consistent with earlier findings of our group (Pardo-Rodriguez et al., 2021a), which applied EMD to HRV signals to examine their interaction with BPts during controlled breathing. That study showed that fast HRV components (like IMF 2) increased Granger causality in the **HRV → brain** direction, while slow HRV components (like IMF 4) enhanced causality in the **brain → HRV** direction. This differentiation between fast and slow components observed in both studies may reflect distinct temporal mechanisms underlying short-latency autonomic responses and slower, integrative regulatory processes during conscious breathing.

One key aspect of these findings is the prominent role of the γ band in the **body↔brain** interactions during **CBTs**. Significant G-causal relationships in the γ band increased notably during both **CBTs**, aligning with prior studies that highlight γ rhythms as crucial for inter-regional brain communication, and extending this role to interactions with peripheral systems. For instance, Herrero et al. (2018) showed that during conscious breathing, γ band coherence increases in regions such as the anterior cingulate and premotor cortex, suggesting involvement in coordinating neural processes during rhythmic activities like breathing. Animal studies have found that respiration modulates the amplitude of γ sub-bands in frontal areas, with respiratory activity phase-locking to neural oscillations, potentially synchronizing brain regions and linking γ rhythms to neural regulation during breathing tasks (Tort et al., 2018, 2025; Zelano et al., 2016). Moreover, γ-band activity has been shown to precede autonomic fluctuations such as heart rate and blood pressure during mental tasks (Umeno et al., 2002), suggesting an anticipatory role in modulating peripheral responses. Pardo-Rodriguez et al. (2021a) also reported increased G-causal relationships in the γ band during a CBT, underscoring the role of high-frequency rhythms in coordinating cortical and autonomic activity. Altogether, these findings suggest that γ oscillations may mediate neuro-autonomic synchronization, potentially bridging central and peripheral systems during conscious breathing. This aligns with broader theories positioning γ band activity as a fundamental mechanism in sensory, cognitive, and physiological regulation (Başar, 2013; Herrmann et al., 2004).

The distribution of G-causal relationships across channels provides valuable insight into the spatial dynamics of **brain↔body** interactions during **CBTs**. During both **CBTs**, the **brain → body** causal relationships were predominantly localized to frontal channels, particularly in the γ band. This frontal predominance suggests that higher-order cognitive and regulatory processes, such as attention and executive control, may play a central role in modulating autonomic responses during controlled breathing (Herrero et al., 2018; Tort et al., 2018). The frontal cortex is involved in regulating both emotional and physiological states, and its engagement in **brain↔body** communication during **CBTs** likely reflects an active closed-loop modulation of autonomic functions in response to the breathing task. This finding resonates with studies showing that prefrontal regions are involved in interoception practices and conscious breathing techniques (Weng et al., 2021), underscoring the role of frontal regions in maintaining control over physiological processes.

Conversely, **body → brain** G-causal relationships shifted toward occipital regions, particularly in the δ, β, and α bands. This may reflect the influence of peripheral physiological signals, such as breathing, on cortical regions involved in sensory processing and integration. This finding mirrors our earlier observation (Pardo-Rodriguez et al., 2021a), where shifts from frontal to occipital regions were linked with changes in breathing patterns and peripheral influences, emphasizing the dynamic and flexible nature of **brain↔body** communication during **CBTs**. Additionally, occipital engagement may relate to the neurophysiological mechanisms underlying slow, deep breathing, which can entrain central autonomic networks and optimize HRV through specific respiratory patterns around 0.1 Hz (Mather and Thayer, 2018; Noble and Hochman, 2019). These breathing-induced shifts may promote coordination between respiratory and sensory processing regions, facilitating the integration of autonomic feedback into cortical areas involved in sensory input and motor coordination.

The widespread increase in BPts synchrony observed during the **CBTs**, particularly in the γ band, but also across slower frequencies, suggests that paced breathing may induce large-scale coordination of cortical rhythms. This pattern aligns with the supramodal entrainment framework (Lakatos et al., 2019), which posits that rhythmic input can drive large-scale cortical oscillatory dynamics beyond sensory-specific regions. **CBTs** may act as a multisensory stimulus—integrating interoceptive (breath), proprioceptive (chest movement), and auditory (cue) inputs—thereby engaging broader sensorimotor and autonomic networks. Supporting this, Tort et al. (2025) has shown that respiration can synchronize neural activity across widespread brain regions, modulating faster oscillations and shaping inter-regional communication.

Interestingly, we observed prominent components at ~0.025 Hz (IMF 4) and ~0.05 Hz (IMF 3), consistent with infra-slow oscillations (ISOs) reported in animal and human studies. Work in rodents has shown serotonin fluctuations at these frequencies organize wake and sleep substate transitions (Cooper et al., 2025). In humans, fMRI and ECoG studies have linked ISOs to dynamic hippocampus-cortex communication during rest and sleep (Mitra et al., 2016). Our findings suggest these slow rhythms may also emerge during active, conscious breathing states such as CBTs. This raises the possibility that slow, controlled breathing could entrain or enhance endogenous ISOs, potentially supporting corticalsubcortical interactions involved in emotional regulation and memory-related processes (Tort et al., 2025).

Finally, EEG band power values may show mixed results across resting states and various tasks in several studies. This study found a decrease in *BPts*_α_ and *BPts*_θ_ at specific brain regions during both **CBTs** compared to **Rest**. Zaccaro et al. (2018) systematic review suggests slow controlled breathing causes decreased θ and increased α power. These results support the notion that controlled breathing can decrease θ activity.

While sex differences in HRV and EEG spectral power are well-documented, the use of relative measures such as BPts and IMFs inherently minimizes their potential influence. Moreover, the controlled environment including a seated posture and adaptation period was designed to reduce sources of variability and ensure more consistent baseline intersubject measures. When analyzing the data by sex, females showed fewer G-causal relationships for **brain↔breathing** during **Rest**, but not during **CBTs**, suggesting that conscious breathing elicits robust cortical-autonomic interactions across sexes.

Future studies should examine pathological models that isolate or alter **brain↔heart** communication pathways to better understand their origin and rule out analysis-related artifacts. Future analyses could include applying EMD to HRV signals to disentangle sympathetic and parasympathetic contributions, as well as assessing the time course of BPts synchronization to the breathing rhythm during task onset.

Taken together, these findings reveal that conscious breathing can modulate cortical-autonomic dynamics through frequency-specific neural mechanisms. By showing that neural oscillations particularly in the γ band dynamically synchronize with autonomic rhythms aligned with the breathing frequency, we provide strong support for the neural basis of respiratory entrainment and highlight the brains active role in flexibly regulating peripheral physiology through distinct spectral pathways. These results contribute to a systems-level perspective on brain-body integration, revealing a dynamic closed-loop interaction essential for maintaining physiological homeostasis and adaptive regulation. This study lays the groundwork for developing breathing-based interventions aimed at enhancing self-regulation, with potential applications in biofeedback, stress management, and improving physiological and mental well-being.

## Data Availability

The raw data supporting the conclusions of this article will be made available by the authors, without undue reservation.
